# Immune response characterization of endometrial cancer

**DOI:** 10.18632/oncotarget.26630

**Published:** 2019-01-29

**Authors:** Yuexin Liu

**Affiliations:** ^1^ Department of Bioinformatics and Computational Biology, The University of Texas MD Anderson Cancer Center, Houston, Texas, USA

**Keywords:** immune response, tumor-infiltrating lymphocyte, prognosis, immunotherapy, endometrial cancer

## Abstract

**Background:**

The comprehensive characterization and prognostic relevance of immune activation in endometrial cancer remain largely unknown.

**Results:**

We systematically reported a subset of endometrioid-type endometrial cancer characterized by multifaceted immune features such as low tumor purity, high leukocyte percentage, and striking *CD8* lymphocytic infiltration with anti-tumor efficacy along with marked upregulation of immunosuppressive gene markers. We also showed that genes whose expression was significantly correlated with better survival were significantly enriched in the immune-related signaling pathways, suggesting that tumor-infiltrating lymphocytes give rise to a favorable prognosis in endometrial cancer. Furthermore, we showed that immune cell recruitment in this subset of tumors is likely due to the transcriptional activation of the STAT1 signaling network.

**Methods:**

We obtained the multi-dimensional genomic data from publicly available databases and correlated them with the four gene expression-based subtypes we recently identified in endometrial cancer. Upstream regulator analysis was used to identify the most significantly enriched transcription regulators and Ingenuity pathway analysis was applied to determine enrichment of signaling pathways in survival-associated genes. Gene set enrichment analysis was performed on the 200-gene T-cell tumor infiltration gene signature comparing Cluster IV with the other three clusters combined. All statistical tests were two-sided, and a *P* value of less than 0.05 is considered significant across all analyses performed.

**Conclusion:**

This study helps to identify patients with immune activation who are likely to benefit from emerging immune checkpoint inhibitors.

## INTRODUCTION

Endometrial carcinoma is the most common gynecologic malignancy, and over 10,470 uterine corpus cancer deaths were estimated in the United States in 2016 [1], an approximately three-fold increase over the past 25 years. The lethality of endometrial cancer is primarily due to disease with advanced stage (III or IV) at the time of diagnosis. Typically, a five-year survival rate of 83–97% is achieved for localized disease, in contrast to 43–67% for stage III disease and only 13–25% for stage IV disease [2]. In addition to standard care, targeted therapies specific to individualized tumors, such as immunotherapy, are needed for advanced-stage patients [3].

Immunotherapy treatments have been responsible for long-lasting responses in different types of cancer [4, 5]. Molecular classification of human cancer represents an important step toward the goal of personalized treatment, and helps to identify patients who would benefit from immunotherapy [6]. Using whole-genome gene expression profiling, multiple studies now have identified a fraction of tumors that are characterized by immunogenic features [7–9]. The Cancer Genome Atlas (TCGA) research network discovered a subset of ovarian carcinoma patients termed “immunoreactive” whose tumors were characterized by upregulation of the T-cell chemokine ligands (*CXCL10* and *CXCL11)* and receptor (*CXCR3*) [7]. Recently, an international consortium reported four consensus molecular subtypes (CMSs) in colorectal cancer, where CMS1 showed strong immune infiltration activation [8]. A claudin-low molecular subtype of high-grade bladder cancer was recently discovered and shown to be immune infiltrated [9]. These patients identified therein are hypothesized to respond to the currently emerging immune checkpoint inhibitors.

Tumor-infiltrating lymphocytes (TILs) have been associated with a survival advantage in different cancer types [10–12]. In patients with endometrial cancer, an increased number of cytotoxic T lymphocytes (CTLs) at the invasive border was reported to be a reliable independent prognostic factor of survival [13]. However, comprehensive characterization and prognostic relevance of immune response in endometrial cancer are largely unknown. Filling this gap in knowledge will enhance our understanding of molecular mechanisms modulating immune surveillance as well as help to identify novel therapeutic strategies for women with endometrial cancer. We previously found patterns with gene expression profiling capable of subdividing endometrioid-type endometrial cancer into four molecular subtypes (I–IV) that have distinct pathologic characteristics and prognostic ability [14]. In contrast to Cluster II with low-grade disease but diminished survival [14], Cluster IV comprised high-grade tumors but exhibited a relatively better prognosis. In the present study we will focus on Cluster IV and perform comprehensive characterization of tumors in this cluster via integrated genomic analyses. Our results show that Cluster IV is uniformly enriched for immune gene signatures and potentially represents an immunoresponsive subtype. We further show that T cell infiltrates exert anti-tumor activity and induce adaptive immune evasion. The prognostic capability of immune activation and a potent transcriptional modulator driving immune response in endometrial cancer are further investigated.

## RESULTS

### Multifaceted characterization of immune response in endometrial cancer

**Figure 1:**
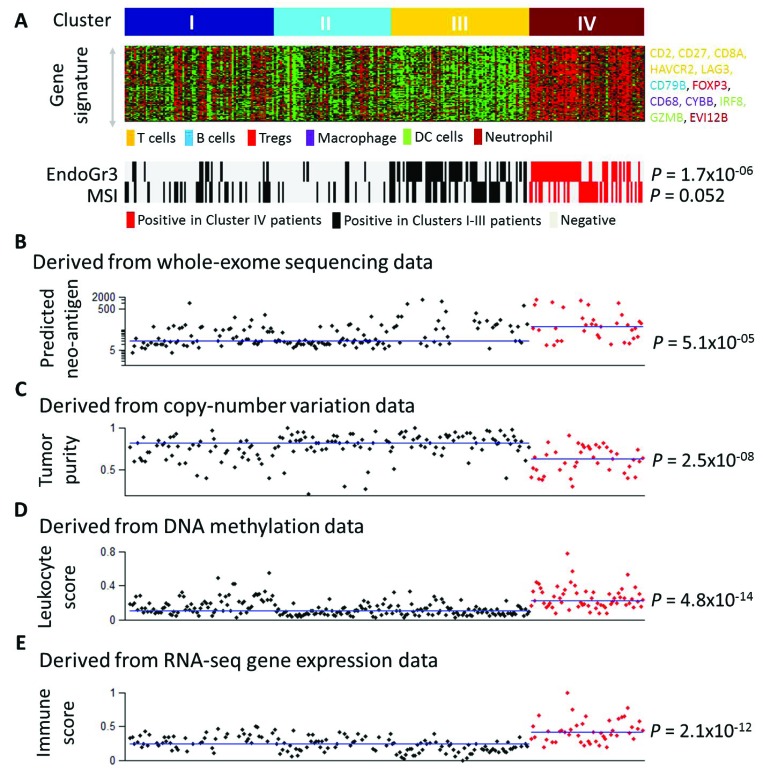
Multifaceted characterization of immune response in endometrial cancer (**A**) Gene signature in Cluster IV and association with grade 3 and MSI tumors. (**B**) Association of Cluster IV tumors with predicted neo-antigens. The neo-antigen burden was derived from whole-exome sequencing data and obtained from ref 15. The Y-axis denotes the number of predicted neo-antigens and is presented in a logarithmic scale. 35 patients in Cluster IV and 156 patients in the other three clusters combined had the neo-antigen data. (**C**) Association of Cluster IV tumors with tumor purity. The tumor purity data derived from copy-number alterations were obtained from ref 17. The Y-axis denotes patient tumor purity. 41 patients in Cluster IV and 152 patients in the other three clusters combined had the tumor purity data. (**D**) Association of Cluster IV tumors with leukocyte score. The leukocyte methylation score was derived from DNA methylation data and obtained from ref 19. The Y-axis denotes patient leukocyte score. 60 patients in Cluster IV and 211 patients in the other three clusters combined had the leukocyte score data. (**E**) Association of Cluster IV tumors with mRNA immune score. The mRNA immune score was derived from RNA-seq gene expression profiling and obtained from ref 20. The Y-axis denotes the patient mRNA immune score. 45 patients in Cluster IV and 150 patients in the other three clusters combined had the mRNA immune score data. In Figure 1B–1E, each dot represents an individual EEC sample. The X-axis is used as “jitter” to simply separate dots and ranges from 1 to 271. The 271 EEC patient samples in Figure 1B–1E were sorted and aligned in the same order as shown in Figure 1A. The horizontal lines in Figure 1B–1E indicate the median values of the corresponding immune parameters (neo-antigens, tumor purity, leukocyte score, and immune score) in the Cluster IV group and in the other three clusters combined group, respectively. Statistical testing of Cluster IV against the other three clusters combined is assessed via the Mann–Whitney test.

We recently identified four gene expression clusters existent in endometrioid-type endometrial cancer with distinct clinicopathologic characteristics and patient outcome [14]. The gene signature associated with Cluster IV consists of genes that represent different immune cell types (Figure 1A). The gene signatures indicative of specific cellular immune populations were obtained from the literature [15]. Cluster IV is significantly enriched with the grade 3 tumors (*P* = 1.7 × 10^−06^, Fisher’s exact test) and over 50% of the Cluster IV cases were microsatellite instable (MSI) (*P* = 0.052) (Figure 1A). Neo-antigens are altered peptides derived from tumor-intrinsic mutant proteins that are presented by the major histocompatibility complex (MHC) molecules and can drive robust antitumor T cell response [16]. Using the predicted neo-antigens in a previous report [15], we next compared Cluster IV to the other three clusters combined, and found that Cluster IV had significantly more neo-antigens (*P* = 5.1 × 10^−05^, Mann–Whitney test, Figure 1B), which indicated the immune responsive capability of this cluster. Moreover, we obtained tumor purity for endometrial cancer patients from the TCGA publication [17] and examined it by molecular subtype. Our results showed that Cluster IV had significantly lower tumor purity (*P* = 2.5 × 10^−08^, Figure 1C). Tumor purity estimated the percentage of tumor cells in a tumor tissue [18], and therefore these data indicated that tumors in Cluster IV contained significantly more non-tumor cellular components such as normal epithelial, stromal, vascular, or immune cells. In addition, we obtained the leukocyte methylation scores for endometrial cancer patients from the PanCanAtlas publication [19] and found that Cluster IV had significantly higher leukocyte methylation scores (*P* = 4.8 × 10^−14^, Figure 1D), suggesting a significantly higher percentage of lymphocyte infiltrate in Cluster IV tumors. A quantitative immune score was calculated from gene expression profiling (mRNA) of curated immune gene signatures to predict the relative level of infiltrating immune cells in the tumor tissue [20]. Using the immune score for endometrial cancer patients provided by this paper [20], we found that Cluster IV had significantly higher mRNA immune scores than the other three subtypes (*P* = 2.1 × 10^−12^, Figure 1E). Collectively, these results from multi-dimensional data platforms (i.e., DNA sequencing, copy number variation, methylation, and mRNA gene expression) concordantly suggest that Cluster IV shows robust and increased lymphocytic infiltrate.

### T cell infiltrates exhibit anti-tumor activity and induce adaptive immune resistance

**Figure 2:**
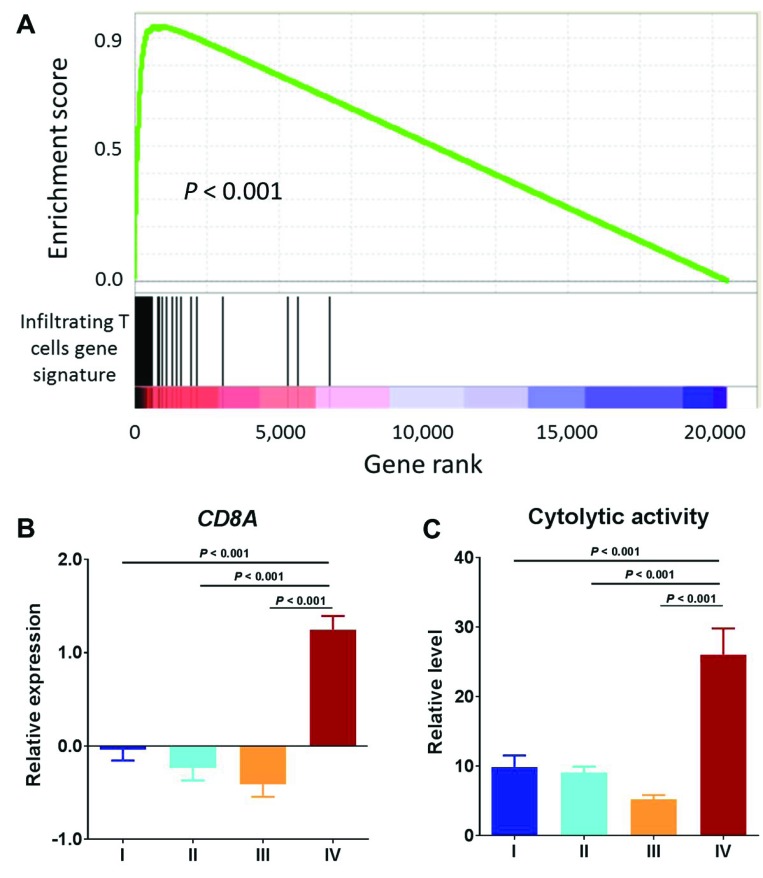
T cell infiltrates exhibit anti-tumor activity (**A**) GSEA of infiltrating T cells gene signature in Cluster IV compared with the other three clusters combined. (**B**) *CD8A* mRNA expression by molecular subtype. (**C**) Relative level of cytolytic activity by molecular subtype.

A recent study identified a 200-gene set that was highly specific to tumor T-cell infiltration [21]. Gene set enrichment analysis (GSEA) of this gene signature in Cluster IV tumors as compared with the other three clusters combined showed striking enrichment in those Cluster IV positively correlated genes, with an enrichment score of as high as over 0.9 (Figure 2A), suggesting an increased T lymphocyte population in the Cluster IV tumors. Consistent with this observation was that expression of the T-cell marker (*CD8A)* was significantly higher in Cluster IV than in all of the other three clusters (*P* < 0.0001, all comparisons, Mann–Whitney test) (Figure 2B). An mRNA-based metric of immune cytolytic activity, defined as average expression of perforin 1 and granzyme A, was devised to quantify anti-tumor immunity [15]. We obtained the cytolytic activity data for TCGA endometrial cancer patients from the literature [15]. Consistent with overexpression of the *CD8A* gene, Cluster IV had the highest level of immune cytolytic activity among all four clusters and thus had higher anti-tumor efficacy, likely due to T- cell infiltration (Figure 2C). In all, these data suggest an increased density of T lymphocyte infiltrates in Cluster IV, which are likely capable of mediating an antitumor effect.

**Figure 3:**
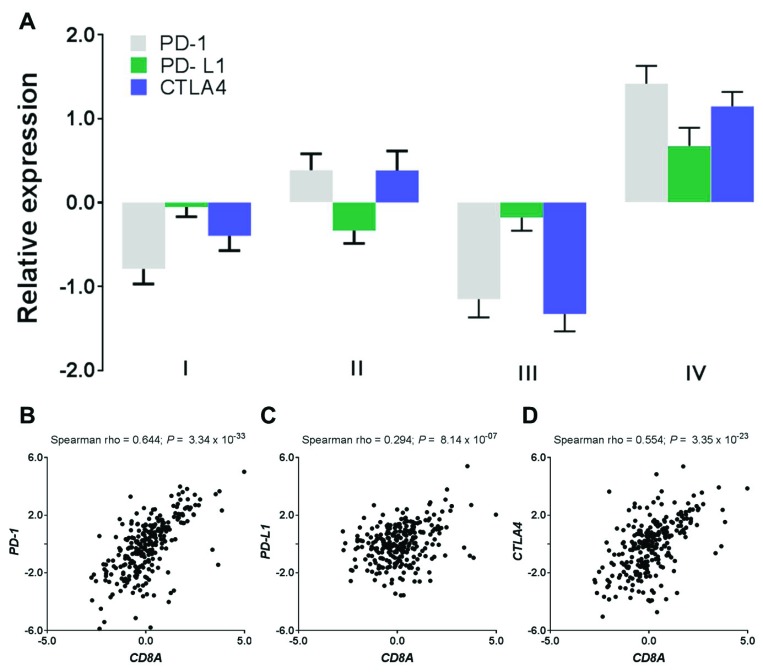
T cell infiltrates induce adaptive immune resistance (**A**) Expression levels of immune checkpoint inhibitors (i.e., *PD-1, PD-L1*, and *CTLA4*) by endometrial subtypes. (**B**–**D**) Expression correlation of T-cell marker (*CD8A*) with the immune checkpoint inhibitors where (B) *CD8A* vs *PD-1*, (C) *CD8A* vs *PD-L1*, and (D) *CD8A* vs *CTLA4*.

Typically a high level of immune infiltration is accompanied by a high level of active immune suppression [9] – a phenomenon termed adaptive immune resistance. We next proceeded to examine expression of a panel of immune checkpoint molecules including T-cell inhibitors and found that these immunosuppressive mediators such as *PD-1* (*P* = 2.52 × 10^−12^, Mann–Whitney test), *PD-L1* (*P* = 6.69 × 10^−05^) and *CTLA4* (*P* = 1.44 × 10^−10^) were significantly and highly expressed in the Cluster IV tumors as compared to the other three clusters combined (Figure 3A). Overexpression of these immune suppression genes rendered resistance to the immune response or evaded the tumors from immune surveillance. Moreover, the expression level of the *CD8A* gene was significantly correlated with *PD-1* (Spearman rho = 0.644, *P* = 3.34 × 10^−33^, Figure 3B), *PD-L1* (Spearman rho = 0.294, *P* = 8.14 × 10^−07^, Figure 3C), and *CTLA4* (Spearman rho = 0.554, *P* = 3.35 × 10^−23^, Figure 3D), suggesting that the adaptive immune resistance to the T-cell response might be induced by T cell infiltrates.

### Prognostic utility of immune activation in endometrial cancer

**Figure 4:**
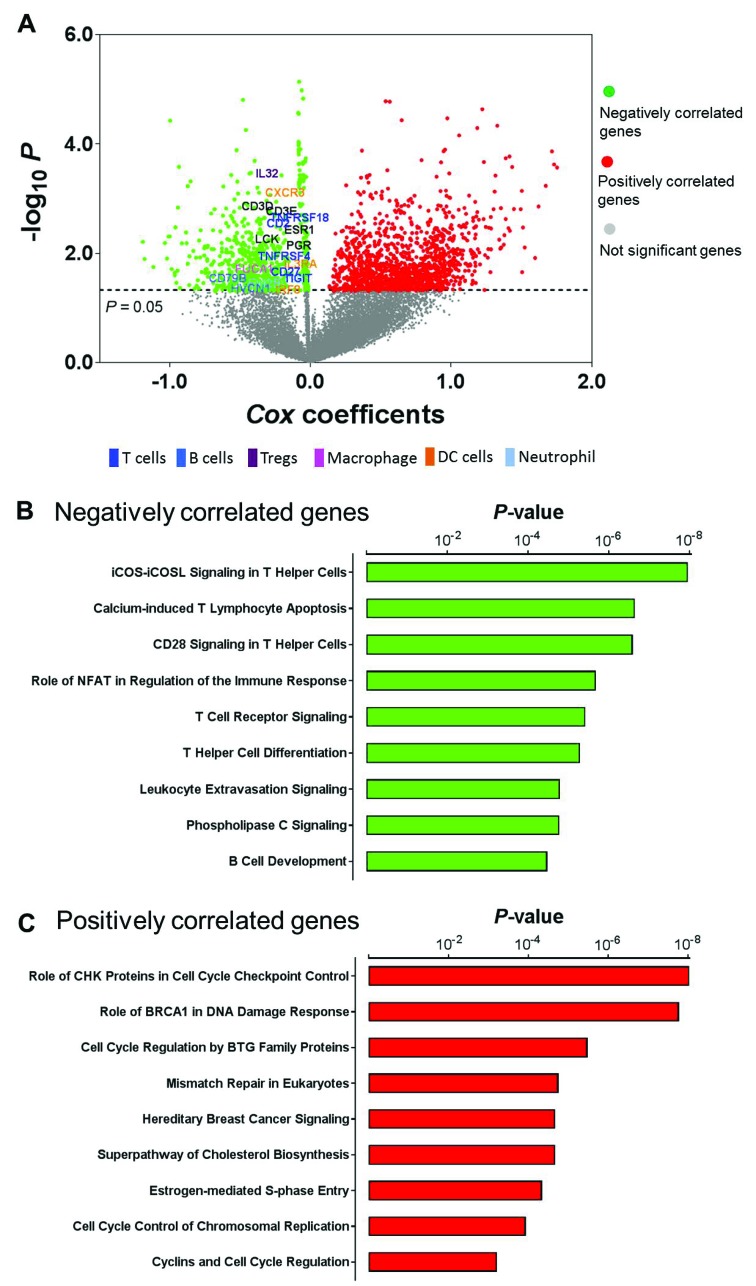
Prognostic utility of immune activation in endometrial cancer (**A**) Correlation of whole-genome gene expression profiling with patient survival in endometrial cancer; green dots indicate genes that were negatively correlated with survival (Cox coefficient < 0, *P* < 0.05), red dots indicate genes that were positively correlated with survival (Cox coefficient > 0, *P* < 0.05), and gray dots indicate genes that were not significantly correlated with survival (*P* > 0.05). (**B**) The most significantly enriched pathways in those negatively correlated genes. (**C**) The most significantly enriched pathways in those positively correlated genes.

We have previously shown that patients in Cluster IV exhibited relatively better survival [14]. Concordance of favorable prognosis and high-level lymphocytic infiltrates in the same tumors suggested that immune activation may partially contribute to tumor progression in endometrial cancer. To systematically interrogate the clinical relevance of immune activation in endometrial cancer, we applied Cox proportional hazards regression modeling to correlate whole-genome gene expression profiling with patient survival. A total of 667 genes were negatively associated with survival (*Cox* coefficient < 0, *P* < 0.05), meaning patients with higher expression of these genes had significantly better survival (Figure 4A, green dots in the plot, Supplementary Table 1). Interestingly, these prognostic genes identified as associated with better survival contain different immune cell signatures [15] including T cell (*CD2, CD27, TIGIT, TNFRSF18, TNFRSF4*), B cell (*CD79B, HVCN1*), Treg cell (*IL32*), macrophage (*FUCA1*), DC cell (*CXCR3, IL3RA, IRF8*), and neutrophil (*EVI2B*). We also found that the T cell activators (*CD3D, CD3E*) and lymphocyte cell-specific protein-tyrosine kinase (*LCK*) were in this gene list. *LCK* is a non-receptor tyrosine kinase commonly associated with T-lymphocyte signaling [22]. As a positive control, this gene set also includes the *ESR1* gene (encoding estrogen receptor alpha) and *PGR* gene (encoding progesterone receptor), which is consistent with a previous report that found that elevated levels of hormone receptors were significantly associated with better survival in endometrial cancer [14]. More prominently, agnostic pathway analysis showed that these negatively correlated genes (*Cox* coefficient < 0) were significantly enriched in immune-related signaling pathways (Figure 4B). Genes belonging to these pathways were provided in Supplementary Table 2. The presence of an immune infiltrate has been shown to be prognostic in cancers, including endometrial cancer [13]. Previous studies have demonstrated a strong correlation between cytotoxic T-cell infiltrate and cancer outcome [12, 13, 23, 24]. Therefore, these data support the notion that tumor-infiltrating lymphocytes result in a favorable prognosis in endometrial cancer patients. On the other hand, 1309 genes were positively associated with survival (*Cox* coefficient > 0, *P* < 0.05, Figure 4A, red dots in the plot, Supplementary Table 1), meaning patients with higher expression of these genes had significantly worse survival. Different from those negatively correlated genes, these positively correlated genes were significantly enriched in signaling pathways that involve cell cycle regulation or DNA damage response (Figure 4C). Genes belonging to these pathways were provided in Supplementary Table 3. It was previously reported that the “mitotic” subgroup in endometrial cancer had activation of cell cycle progression and was significantly associated with worse survival [17]. Among these survival-related genes, five genes (*EPHB2, FBP1, NLRC3, PPP2R3A*, and *TRIM46*) were included in a previously reported 9-gene signature for predicting endometrial cancer patient survival [25].

### STAT1 is the key regulator of immune activation in endometrial cancer

**Figure 5:**
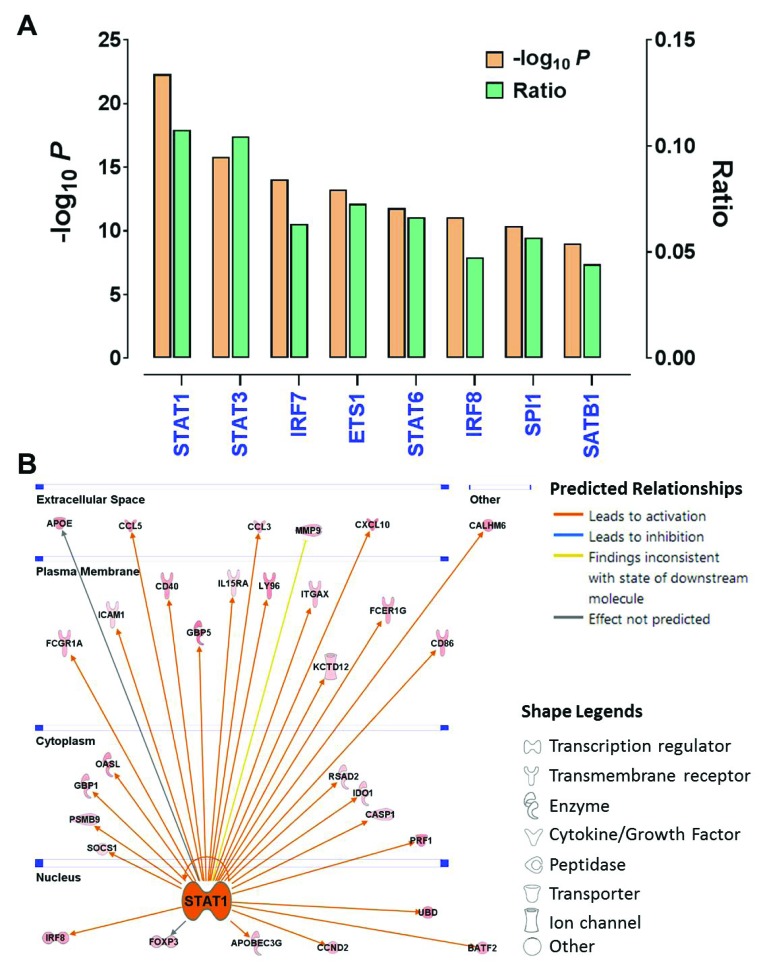
STAT1 is the key regulator of immune activation in endometrial cancer (**A**) The *P* values and ratios of the top transcription factors enriched in the Cluster-IV associated gene signature were identified by IPA. The ratio indicates the number of predicted targets to the total genes included in the signature gene set. (**B**) The gene network showing the predicted relationship between STAT1 and downstream targets was generated by IPA. The locations (i.e., nucleus, cytoplasm, plasma membrane and extracellular space) and biological functions (indicated by the node shapes) of these targets also were shown.

Having shown that Cluster IV is an immunoresponsive subtype, we next sought to investigate the underlying mechanism that leads to tumor immune activation in this cluster. We first used Ingenuity Pathway Analysis to analyze the gene signature that is indicative of the Cluster IV tumors (Figure 1A), which revealed that *STAT1* was the most affected transcriptional factor in the upstream regulator rankings based on its *P* value (*P* = 4.78 × 10^−23^) with overlapping dataset genes. Over 10% of the signature genes were predicted to be the *STAT1*’s targets (Figure 5A). Interestingly, the other two members (*STAT3* and *STAT6*) in the STAT gene family also were in this significantly enriched transcription factor list. The *STAT1’s* downstream genes displayed a well-interconnected regulatory system with *STAT1* as a central node (Figure 5B). The gene expression changes are consistent with the predicted relationship found in the literature across most targets. *STAT1* is consistently predicted to lead to activation of most of the downstream targets indicated by the orange connecting lines. For instance, the transcriptional expression of such downstream targets as *CXCL10* [26], *CD40* [27], and *FOXP3* [28] was previously reported to be activated by phosphorylation of STAT1. These data suggest that *STAT1* is a key modulator regulating the expression pattern of the Cluster IV-associated gene signature, and thus driving the phenotype of Cluster IV tumors. Consistent with previous reports, STAT1 was reported to induce immunogenicity in head and neck cancer [29], and silencing of STAT1 expression led to immune evasion in melanoma cells [30].

## DISCUSSION

By genomic analysis of a comprehensive dataset including multi-dimensional data platforms, we have demonstrated that one of the discovered subtypes in our previous study [14], Cluster IV, is characterized by a high amount of neo-antigens, low tumor purity, and a gene signature of T-cell infiltrate along with marked upregulation of immune suppressive gene markers. We further showed that immune cell recruitment in this subset of tumors is likely due to the transcriptomic regulation of a key transcription factor, *STAT1*. As a consequence of cytotoxic T-cell infiltration, the genes identified to be correlated with better survival in endometrial cancer are significantly enriched in signal pathways that involve immune response.

Despite upregulation of the immunosuppressive genes, we also found substantial increases in the cytotoxic T cell marker and cytotoxic differentiation markers in Cluster IV, suggesting that the degree of adaptive immune resistance in these tumors may be insufficient to fully suppress CD8 T-cell cytotoxicity [31]. Collectively, our data suggest a complex interaction between the antigenic landscape of Cluster IV tumors and the immune response. Interestingly, Cluster IV was significantly enriched with the TCGA *POLE* cluster which had ultra-high mutation rates associated with *POLE* mutations [14]. Consistent with our study, somatic mutations in the *POLE* gene were associated with immune cell infiltration [6], eliciting an antitumor immune response in endometrial cancer patients [32]. Moreover, PD-1 and PD-L1 also were significantly overexpressed in Cluster IV tumors. Of note, response to anti-PD-L1 antibody MPDL3280A has been shown to correlate with the expression of PD-L1 in tumor-infiltrating immune cells but not in tumor cells across a range of cancers [33]. Although there are no prior studies of immunotherapy performed specifically for patients with endometrial cancer, there is one ongoing clinical trial (NCT01876511) of the PD-1 antibody nivolumab in all cancers with MSI status. Although Clusters III and IV were both enriched with MSI-high tumors (Figure 1A), our present study shows that patients in these two clusters exhibited quite different immune characteristics, suggesting that patients in Cluster IV but not in Cluster III are excellent candidates for immune checkpoint inhibitor therapy targeting the PD-1 pathway. These data are consistent with a recent study that showed that the role of immunotherapy was different in endometrial cancer patients with either hereditary or sporadic MSI-H tumors [34].

The number of predicted neo-antigens has been positively associated with favorable clinical outcomes for multiple tumor types [35], as well as with the response to immune checkpoint inhibition in melanoma [4, 36] and non-small-cell lung cancer [5]. There is a significant correlation between predicted neo-antigen burden and the number of somatic mutations in this cohort, which is consistent with a previous study [9]. Although Cluster IV had a higher level of immune infiltration than Cluster III, there was no significant difference in predicted neo-antigen burden between these two clusters; both shared the similar clinical features of being high-grade and high-stage diseases [14]. Therefore, immune activation in Cluster IV tumors did not appear to be fully explained by the predicted neo-antigen burden.

Our study is not without limitations. Although we showed overexpression of the immune cell markers at the mRNA level, the protein levels of these biomarkers are not known. An immunohistochemistry assay is necessary to further corroborate these associations. Furthermore, the retrospective nature of our study meant that we were unable to investigate the antigen response of T cells in Cluster IV patients. This and other functional analyses will require prospective investigation. Future work also includes identification of a classifier or some genetic features for recognizing tumors in Cluster IV. Nevertheless, our data demonstrated that immune cell recruitment due to activation of STAT1 signaling axis contributes to a favorable prognosis for endometrial cancer and that Cluster IV tumors are potential candidates for immune checkpoint inhibitor therapy.

## MATERIALS AND METHODS

### Patient samples

Gene expression cluster assignment and genomic data regarding molecular subtyping of endometrioid-type endometrial cancer were from our recent work [14]. RNA-seq gene expression data and clinicopathologic characteristics of a total of 271 endometrioid-type endometrial cancer patients after exclusion of serous cases were obtained from the TCGA data portal (https://portal.gdc.cancer.gov/) in March 2013. The mean age at diagnosis was 62.0 (range: 33 to 90 years). Consensus clustering of this cohort of patients identified four transcriptome subtypes, resulting in 78 in Cluster I, 61 in Cluster II, 72 in Cluster III and 60 in Cluster IV [14]. Patient overall survival is defined as the interval from the date of initial surgical resection to the date of last known contact (censored) or death. Access to the TCGA database was approved by the National Cancer Institute. MD Anderson Cancer Center waived the requirement for ethical approval of this analysis because the registry contains only de-identified data. Written consent was obtained from all living patients.

### Genomic data sets

Tumor purity estimates the fraction of tumor cells in tumor tissues and is determined from somatic DNA copy number alterations [18]. The tumor purity data for endometrial cancer patients were obtained from this literature [17]. The leukocyte methylation scores were derived from DNA methylation data, and used to estimate the proportion of a heterogeneous tumor sample that consists of leukocytes [19]. We downloaded the leukocyte methylation scores from Synapse (https://www.synapse.org). The immune scores from Yoshihara, *et al.* [20] were calculated from gene expression profiling (mRNA) of curated immune gene signatures to predict the relative level of infiltrating immunes in the tumor tissue. Cytolytic activity was calculated as the average value of *PFR1* (encoding perforin 1) mRNA expression and *GZMA* (encoding granzyme A) mRNA expression to quantify anti-tumor immunity [15]. The neo-antigen burden was predicted from the whole-exome sequencing data. The cytolytic activity data and predicted neo-antigens for endometrial cancer patients were both obtained from Rooney, *et al.* [15]. The immune gene signatures used to describe immune cell types were derived by Rooney, *et al.* [15]. The published 200-gene T-cell tumor infiltration gene signature was identified by Johnston, *et al.* [21].

### Gene expression data analysis, pathway analysis, upstream regulator analysis, and GSEA

The gene expression data obtained from TCGA were first median centered and then log2 transformed across the entire cohort. The gene signature that is associated with Cluster IV was identified previously [14] and upstream regulator analysis was used to identify transcription regulators enriched in this gene set. Pathway analysis (Ingenuity Pathway Analysis, IPA) was applied to identify enrichment of signaling pathways in survival-associated genes. Gene set enrichment analysis (GSEA) was performed to determine whether the 200-gene T-cell tumor infiltration gene signature [21] showed statistically significant association with the Cluster IV phenotype. Genes were ranked in descending order on the basis of the signal-to-noise ratios of genes comparing Cluster IV with the other three clusters combined. The enrichment score was calculated by walking down the ranked gene list and increases when a gene was in the pre-defined gene set and decreased when it was not [37].

### Statistical analysis

We used the nonparametric Mann–Whitney test for all comparisons of continuous data and the Spearman correlation coefficient (*rho*) to analyze correlation between different variables. Fisher’s exact test was used to compare the tumor grade or MSI status between Cluster IV and the other three clusters combined. Survival analysis on continuous variables such as gene expression was performed using a Cox proportional hazards model to derive coefficients and *P* values as determined by the Wald test. The statistical significance for both pathway analysis and upstream regulator analysis was assessed via Fisher’s exact test. All statistical tests were two-sided, and a *P* value of less than 0.05 is considered significant across all analyses performed. Except where indicated, statistical tests were unadjusted. Statistical analyses were performed using the following scientific software: Matlab version 8.4 (MathWorks, Inc., Natick, MA), SPSS version 18 (SPSS Inc., Chicago, IL), and GraphPad Prism, version 6 (GraphPad Software, Inc., La Jolla, CA).

## SUPPLEMENTARY MATERIALS TABLES




