# Supplementary material for: Immune response characterization of endometrial cancer
Source: Oncotarget. 2019 Jan 29;10(9):982–92. doi: 10.18632/oncotarget.26630 (PMC6398181; doi:10.18632/oncotarget.26630)
Supplement: Supplementary file 1 [file oncotarget-10-982-s001.pdf]

Immune response characterization of endometrial cancer

SUPPLEMENTARY MATERIALS

Supplementary Table 1: Details of genes that were significantly correlatd with survival ($P < 0.05$). See Supplementary_Table_1

Supplementary Table 2: Details of the most significantly enriched pathways in those negatively correlated genes (Supplementary to Figure 4B)

Ingenuity canonical pathways	−log(*p*-value)	Molecules
iCOS-iCOSL Signaling in T Helper Cells	7.96	CD3E,PTEN,HLA-DQA1,HLA-DOB,NFATC1,IL2RG,LCP2,INPP5D,HLA-DMA,CD3D,LCK,HLA-DMB,ITK,CD4,PTPRC,CD247,ZAP70
Calcium-induced T Lymphocyte Apoptosis	6.65	CD3E,HLA-DMA,HLA-DQA1,LCK,CD3D,HLA-DMB,PRKCB,CD4,HLA-DOB,PRKCH,CD247,ZAP70
CD28 Signaling in T Helper Cells	6.6	CD3E,HLA-DQA1,HLA-DOB,NFATC1,LCP2,WAS,HLA-DMA,CD3D,LCK,HLA-DMB,ITK,CD4,PTPN6,PTPRC,CD247, ZAP70
Role of NFAT in Regulation of the Immune Response	5.69	CD3E,PLCB2,HLA-DQA1,CD79B,BTK,HLA-DOB,NFATC1,LCP2,GNB5,HLA-DMA,CD3D,LCK,HLA-DMB,ITK,CD4,GNA14,CD247,ZAP70
T Cell Receptor Signaling	5.42	CD3E,BTK,NFATC1,LCP2,LCK,CD3D,ITK,BMX,CD4,PTPRC,CD247,PTPN7,ZAP70
T Helper Cell Differentiation	5.29	STAT4,HLA-DMA,HLA-DQA1,STAT6,HLA-DMB,IL4R,HLA-DOB,TBX21,IL2RG,IL12RB1,IL18R1
Leukocyte Extravasation Signaling	4.79	MMP25,NCF4,RHOH,SPN,ITGAL,MMP11,BTK,NCF1,RAC2,RASSF5,WAS,ICAM3,SELPLG,ITK,BMX,PRKCB,PRKCH,ARHGAP9
Phospholipase C Signaling	4.78	CD3E,RHOH,PLCB2,CD79B,BTK,NFATC1 ,PLA2G4C,LCP2,ARHGEF6,GNB5,CD3D, LCK,JMJD7-PLA2G4B,ITK,MYL3,PRKCB, PRKCH,CD247,ZAP70,ARHGEF18
B Cell Development	4.48	SPN,HLA-DMA,HLA-DQA1,CD79B,HLA-DMB,HLA-DOB,PTPRC

Supplementary Table 3: Details of the most significantly enriched pathways in those positively correlated genes (Supplementary to Figure 4C)

Ingenuity canonical pathways	−log(*p*-value)	Molecules
Role of CHK Proteins in Cell Cycle Checkpoint Control	8.04	E2F5,CDK2,CDC25A,E2F1,CLSPN,MDC1,PPP2R3A,PPP2R2A,RAD1,RFC1,E2F2,RFC2,PPP2R5D,RPA1,PPP2CB,RFC4,CHEK1
Role of BRCA1 in DNA Damage Response	7.78	E2F5,E2F1,FAAP24,BARD1,MDC1,SMARCD2,SMARCC1,BRIP1,RFC1,TOPBP1,FANCE,MSH6,E2F2,SMARCD3,RFC2,RPA1,RBL1,RFC4,MSH2,CHEK1
Cell Cycle Regulation by BTG Family Proteins	5.49	E2F5,CDK4,E2F2,NOCT,CDK2,PPP2CB,PPP2R5D,E2F1,PPP2R3A,CNOT7,PPP2R2A
Mismatch Repair in Eukaryotes	4.76	RFC1,EXO1,MSH6,RFC2,RPA1,RFC4,MSH2
Hereditary Breast Cancer Signaling	4.68	HMGCS1,IDI1,DHCR24,MVK,FDPS,ACAT2,CYP51A1,FDFT1,DHCR7
Superpathway of Cholesterol Biosynthesis	4.68	KRAS,CDK4,E2F1,FAAP24,PIK3C2B,BARD1,SMARCD2,SMARCC1,POLR2H,RFC1,PIK3CA,POLR2D,FANCE,MSH6,SMARCD3,RFC2,RPA1,HDAC4,RFC4,MSH2,CHEK1
Estrogen-mediated S-phase Entry	4.35	E2F5,CDK4,E2F2,SKP2,CDK2,CDC25A,E2F1,RBL1
Cell Cycle Control of Chromosomal Replication	3.94	CDK4,MCM7,MCM2,CDK2,ORC2,RPA1,MCM6,MCM4
Cyclins and Cell Cycle Regulation	3.22	E2F5,CDK4,CDK2,CDC25A,E2F1,PPP2R3A,PPP2R2A,SKP2,E2F2,PPP2R5D,CDKN2B,PPP2CB,HDAC4
